# Procedural Costs of Robot-Assisted and Laparoscopic Ventral and Incisional Hernia Repair. A Propensity-Score Matched Nationwide Database Study

**DOI:** 10.3389/jaws.2025.15464

**Published:** 2025-11-04

**Authors:** Nadia A. Henriksen, Mette Willaume Christoffersen, Mads Marckmann, Kristian K. Jensen

**Affiliations:** ^1^ Digestive Disease Center, Bispebjerg Hospital, Copenhagen, Denmark; ^2^ Department of Surgery, Zealand University Hospital, Koege, Denmark; ^3^ Department of Surgery and Transplantation, Rigshospitalet, Copenhagen, Denmark

**Keywords:** incisional hernia, umbilical hernia, readmission, length of stay, robotic surgery

## Abstract

**Background:**

The utilization of the robotic platform for ventral hernia repair is increasing, however is facing criticism for perceived high costs. This study aimed to compare the procedure-specific costs of robot-assisted ventral or incisional hernia repair with laparoscopic repair.

**Methods:**

This propensity score-matched nationwide database study included patients undergoing primary ventral and incisional hernia repair from 2017 to 2022. A total of 554 patients undergoing robot-assisted repair were matched 1:1 with patients undergoing laparoscopic repair by the confounding variables of age, type of hernia (primary ventral/incisional), and horizontal defect size. The primary outcome was the total cost per procedure in Euros including robotic/laparoscopic approach, mesh, tackers, length of stay, readmission, and operative reintervention. The price of obtaining laparoscopic and robotic systems was not included.

**Results:**

The length of stay was significantly shorter, and readmission rate was significantly lower for robot-assisted repairs (0.5 days and 7.0%) than for laparoscopic repairs (1.2 days and 12.5%), P < 0.001 and P = 0.003, respectively. The mean procedural cost of an incisional hernia repair was significantly reduced with the robot-assisted approach (1,533 Euros (sd: 1,584)) compared to the laparoscopic approach (2,077 Euros (sd: 1,840), *P* = 0.002). Multivariable linear regression analysis confirmed that robotic ventral hernia repair was independently associated with decreased overall costs (coeff −682.1, CI −1,331.5 - −32.6, *P* = 0.040).

**Conclusion:**

For primary ventral hernias, the mean procedural costs of a robot-assisted and a laparoscopic repair are comparable, but for incisional hernia repairs the mean procedural cost is decreased with a robot-assisted approach.

## Introduction

Primary ventral and incisional hernia repairs are common elective procedures that can be done through either open or minimally invasive methods. Open repairs have a notable risk of wound-related complications, which can lead to higher short-term patient morbidity, increased hospital costs, and a greater long-term risk of hernia recurrence [1–3].

Laparoscopic ventral hernia repair was introduced three decades ago and proved to be superior to open repair in terms of decreasing wound complications and suggestively even reducing recurrence rates [4–8]. Laparoscopic ventral hernia repair is conventionally performed as an IPOM (intraperitoneal onlay mesh) technique, where an intraperitoneal mesh is fixated to the abdominal wall with tackers and/or transfascial sutures [9]. Overall, the IPOM approach has good long-term results, but rare, severe complications, such as intestinal adhesions to the intraperitoneal mesh, as well as acute and chronic pain due to traumatic mesh fixation may occur [7]. Meanwhile, the increased access to the robotic platform in high income countries has facilitated the spread of more technically advanced minimally invasive procedures with mesh placement outside the peritoneal cavity avoiding traumatic fixation [10, 11].

Compared to open approaches robot-assisted ventral hernia repair has proven to be superior in terms of less postoperative pain, faster recovery, and fewer wound complications [12–16]. As the robotic platform makes it easier to avoid intraperitoneal mesh and the use of tacks for fixation, there is less postoperative pain, shorter length of stay, and lower risk of readmission compared to laparoscopic IPOM [17–19]. Nonetheless, opponents of the robotic approach argue that it is too costly with prolonged operating times and insufficient data to demonstrate improved patient-related outcomes [20, 21]. Conversely, coated meshes for intraperitoneal use and tacker devices for laparoscopic ventral hernia are also expensive. Thus, if length of stay and readmission rate increase, the costs of a laparoscopic approach may surpass that of a robot-assisted approach.

The hypothesis of the present study was that the procedural costs of robot-assisted primary ventral or incisional hernia repair are equal to or lower compared to laparoscopic repair due to shorter length of stay, lower risk of readmission, and less expensive materials. The aim of this study was to compare the procedure-specific costs of a robot-assisted ventral or incisional hernia repair to a laparoscopic repair, including expenses related to readmissions and reoperations within 90 days postoperatively.

## Methods

### Patient Cohort

Since 2007, all hernia repair patients in Denmark have been registered in the Danish Hernia Database without patient consent. Surgeons record details such as hernia type, defect size, mesh type, and fixation method. In 2017, smoking and BMI data were added. The National Patients Registry logs all patient interactions with the healthcare system, including Charlson Comorbidity Index scores, hospital stay duration, readmissions, and reoperations within 90 days. Data for this study were obtained by merging these two databases using each patient’s unique identification number [22]. Patients were included from Jan 1st 2017 to December 31st 2022. Patients undergoing elective primary ventral or incisional hernia repair by either robot-assisted or laparoscopic approach were included. Converted procedures were not included.

### Cost Analysis

The study’s primary outcome was the total mean cost per procedure in Euros. A cost analysis compared laparoscopic and robot-assisted procedures, assuming similar staff numbers and comparable anesthesia and basic instrument costs. The cost variables included were: robot-assisted approach, laparoscopic approach, extra ports (if more than three used), mesh, tackers, hospital stay length, readmission, and surgical reintervention.

The cost of a robot-assisted procedure included the instrument arm drape, three instrument cannulas, obturators, cannula seal, tip covers, and instruments such as monopolar curved scissors, fenestrated bipolar forceps, and a mega suture cut needle driver. The cost of a laparoscopic procedure included ports, scissors or energy devices, and a smoke evacuation tube (Supplementary Table). The costs for buying and maintaining the laparoscopic and robotic systems were not included.

The daily cost for postoperative hospital stay and readmissions within 90 days was calculated using the national Diagnosis Related Grouping (DRG) rate, which measures hospital costs associated with procedures. The same system was used to estimate the cost of reoperation, applying the DRG rate “Other operations and treatments on digestive organs without complicated secondary diagnoses” to avoid overestimation. The same price was applied for every surgical reintervention.

### Statistics

To reduce heterogeneity, robot-assisted primary ventral and incisional hernia repairs were propensity-score matched in a 1:1 ratio with patients undergoing laparoscopic repair. Patients were matched by age, type of hernia (primary ventral/incisional), and horizontal defect size using nearest neighbor matching with a caliper of 0.05. Numerical variables were reported as mean (standard deviation, SD) or median (range) and compared across the two groups using Student’s t-test or Mann–Whitney U test where appropriate. Categorical variables were reported as n (%) and compared across groups using the Chi-squared test. A multiple linear regression analysis was performed to examine the association between mean total cost per procedure and the confounding variables. Additionally, a subgroup analysis of patients undergoing either primary or incisional hernia repair was conducted to evaluate the costs for the specific hernia types.

P-values <0.05 were considered statistically significant. The data analysis was performed using R software version 4.0.2 (R Foundation for Statistical Computing, Vienna, Austria).

This study was approved by the Danish Data Protection Agency (ref. P-2021-58). A protocol was made before study start but was not published online. The study was registered at ClinicalTrials.gov identifier: NCT06232148. The study is reported according to the STROBE reporting guidelines for observational studies [23].

## Results

**FIGURE 1:**
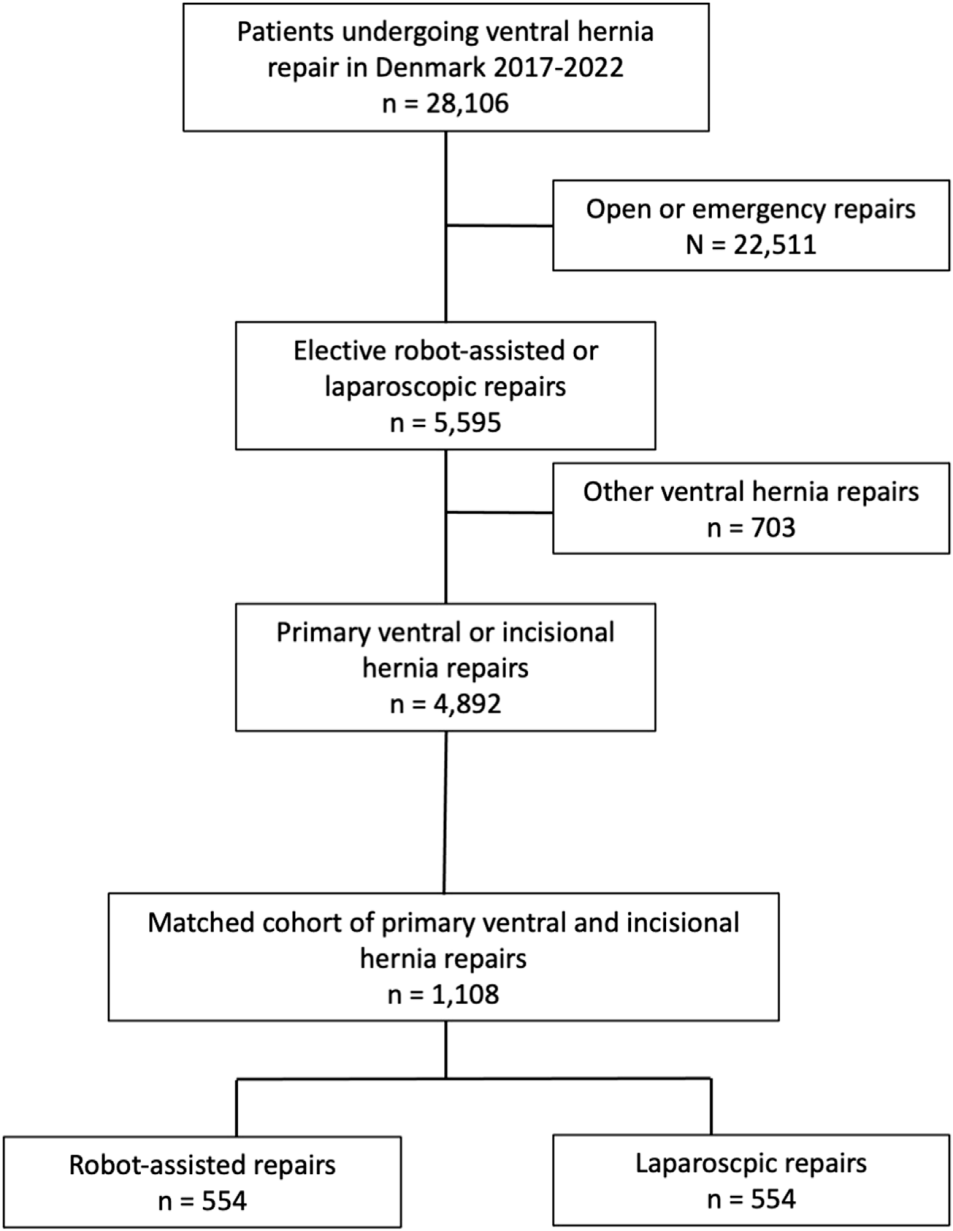
Flowchart of patient inclusion.

A total of 554 patients undergoing robot-assisted primary ventral or incisional hernia repair were matched with 554 patients undergoing laparoscopic repair (Figure 1; Table 1). A total of 35.7% (198/554) of the patients underwent incisional hernia repair and 73.5% (407/554) had a defect width <4 cm. There were no significant differences between the two groups regarding age, sex, smoking status, BMI, and Charlson Comorbidity Index score (Table 1).

**TABLE 1:** Demographics of patients undergoing robot-assisted and laparoscopic ventral and incisional hernia repair.

Variables		Robot-assisted repair (n = 554)	Laparoscopic repair (n = 554)	P
Age (years)	<45	93 (16.8)	117 (21.1)	0.236
	45–60	196 (35.4)	198 (35.7)	
	>60–75	216 (39.0)	198 (35.7)	
	>75	49 (8.8)	41 (7.4)	
Sex	female	239 (43.1)	249 (44.9)	0.586
Smoking	Yes	91 (16.4)	69 (12.5)	0.073
Charlson Comorbidity Index	0	307 (55.4)	305 (55.1)	0.430
	1	92 (16.6)	79 (14.3)	
	>1	155 (28.0)	170 (30.7)	
BMI (kg/m^2^)	mean [range]	30.2 [19.7, 50.8]	30.3 [12.5, 51.6]	0.769
	missing	257	182	
categorized (kg/m^2^)	<30	158 (53.2)	192 (51.6)	0.450
	30–35	89 (30.0)	75 (20.2)	
	>35	50 (16.8)	105 (28.2)	
Type of hernia	Primary ventral	356 (64.3)	356 (64.3)	100
	Incisional	198 (35.7)	198 (35.7)	
Vertical defect size (cm)	mean (sd)	4.5 (5.1)	3.9 (5)	0.064
Horizontal defect size (cm)	mean (sd)	4 (4.6)	3.6 (4.7)	0.238
categorized (cm)	<4	407 (73.5)	407 (73.5)	100.000
	4–8	127 (22.9)	127 (22.9)	
	>8	20 (3.6)	20 (3.6)	
Year of surgery	2017	30 (5.4)	204 (36.8)	<0.001
	2018	88 (15.9)	156 (28.2)	
	2019	108 (19.5)	61 (11.0)	
	2020	102 (18.4)	66 (11.9)	
	2021	146 (26.4)	33 (6.0)	
	2022	80 (14.4)	34 (6.1)	
Mesh position	Preperitoneal	109 (19.7)	128 (23.1)	<0.001
	Retromuscular	419 (75.6)	0 (0.0)	
	Intraperitoneal	26 (4.7)	426 (76.9)	
Mesh fixation method	Glue and tackers/clips	5 (0.9)	0 (0.0)	<0.001
	Glue	22 (4.0)	4 (0.7)	
	Self fixating mesh	377 (68.1)	12 (2.2)	
	Tackers	8 (1.4)	373 (67.3)	
	Glue and suture	4 (0.7)	0 (0.0)	
	Clips	0 (0.0)	3 (0.5)	
	Suture	49 (8.8)	69 (12.5)	
	Tackers and suture	9 (1.6)	78 (14.1)	
	None	76 (13.7)	11 (2.0)	
	Other	4 (0.7)	4 (0.7)	
Defect closure	*Yes*	538 (97.1)	406 (73.3)	<0.001
Length of stay (days)	mean [range]	0.5 [0, 32]	1.2 [0, 29]	<0.001
	missing	22	15	
Readmission, n (%)	Yes	39 (7.0)	69 (12.5)	0.003
Reoperation, n (%)	Yes	11 (2.0)	21 (3.8)	0.106

SD, Standard deviation; BMI, Body Mass Index.

In robot-assisted repairs, the mesh was most often placed in the retromuscular position 75.6% (419/554), and most frequently this was a self-fixating mesh 68.1% (337/554). For the laparoscopic repairs, the mesh was placed in the intraperitoneal position in 76.9% (426/554) and fixated with tackers in 67.3% (373/554) of the cases. In the robotic group, 4.9% (27/554) of the patients underwent a transversus abdominis release (TAR) as opposed to 0% in the laparoscopic group.

Length of stay was significantly shorter after a robot-assisted repair compared to a laparoscopic repair (mean 0.5 days vs. 1.2 days, P < 0.001). The readmission rate was significantly lower for robot-assisted repairs 7.0% (39/554) compared with laparoscopic repairs 12.5% (69/554), P = 0.003. There were no significant differences in the rate of surgical reintervention after robot-assisted or laparoscopic repair (2.0%, 11/554 vs. 3.8%, 21/554), P = 0.106 (Table 1).

The mean cost of basic equipment for a robot-assisted approach was significantly higher compared to laparoscopic approach (865.3 vs. 471.6 Euros, P < 0.001). The mean costs of mesh and tack devices were significantly higher in the laparoscopic group (415 Euros and 153 Euros) than in the robotic group (191 Euros and 10 Euros), P < 0.001, respectively. Mean costs of readmission and reoperation were not significantly higher in the laparoscopic group (425 Euros and 145 Euros) than in the robot-assisted group (63 Euros and 95 Euros), P = 0.257 and P = 0.277, respectively (Table 2). The mean total cost of all robot-assisted primary ventral and incisional hernia repairs was less after robotic compared to laparoscopic repair (1,326.2 vs. 1,990.1 Euros, *P* = 0.045). Multivariable linear regression analysis confirmed that robotic ventral hernia repair was independently associated with decreased overall costs (coeff −682.1, CI −1,331.5 - −32.6, *P* = 0.040, Table 3). In addition, defect size between 4 and 8 cm was associated with increased costs.

**TABLE 2:** Mean total cost per patient for robot-assisted and laparoscopic primary ventral and incisional hernia repair.

Variables		Robot-assisted repair (n = 554)	Laparoscopic repair (n = 554)	P
Costs (Euros)
Robotic approach	Mean (sd)	865.3 (0)	0 (0)	<0.001
Laparoscopic approach	Mean (sd)	0 (0)	471.6 (0)	<0.001
Mesh	Mean (sd)	153 (86)	415 (130)	<0.001
Tackers	Mean [sd]	10 (48)	191 (91)	<0.001
Length of stay	Mean (sd)	140 (557)	344 (677)	<0.001
Readmission	Mean (sd)	63 (365)	425 (7,514)	0.257
Reoperation	Mean (sd)	95 (730)	145 (785)	0.277
Total	Mean (sd)	1,326.2 (1,189.8)	1,990.8 (7,720.8)	0.045

**TABLE 3:** Multivariable analysis of factors associated with total costs for patients undergoing primary ventral or incisional hernia repair.

Variables		Coefficient	95% CI	P
Age (years)		21.4	[-4.95; 47.8]	0.112
Sex	Female	Ref		
	Male	267.0	[-398.7; 932.7]	0.432
Charlson Comorbidity Index	0	Ref		
	1	68.2	[-881.9; 1,018.3]	0.888
	>1	762.7	[-51.1; 1,576.5]	0.067
Horizontal defect, cm	<4	Ref		
	4–8	1,053.3	[250.4; 1856.1]	0.010
	>8	362.8	[-1,454.2; 2,179.9]	0.696
Type of surgical approach	Laparoscopic	Ref		
	Robot-assisted	−682.1	[-1,331.5;-32.6]	0.040
Type of hernia	Incisional	Ref		
	Primary	262.6	[-488.5; 1,013.6]	0.493

### Primary Ventral and Incisional Hernia Repair Subgroup Analysis

A total of 712 patients in the study cohort underwent primary ventral hernia repair, either laparoscopic (n = 356) or robot-assisted (n = 356). Cost analysis of this subgroup found that the mean cost of robot-assisted repair was comparable to laparoscopic repair (1,211 Euros vs. 1,943 Euros, *P* = 0.149, Table 4). Multivariable linear regression analysis found no association of approach with the total costs of the procedure (robotic coeff −714.3, CI −1705.8 – 277.3, *P* = 0.158) for primary ventral hernia repair.

**TABLE 4:** Mean total cost per patient for robot-assisted and laparoscopic primary ventral hernia repair.

Variables		Robot-assisted repair (n = 356)	Laparoscopic repair (n = 356)	P
Costs (Euros)				
Robotic approach	Mean (sd)	865.3 (0)	0 (0)	<0.001
Laparoscopic approach	Mean (sd)	0 (0)	471.6 (0)	<0.001
Mesh	Mean (sd)	139 (54.6)	395 (135.1)	<0.001
Tackers	Mean [sd]	7 (38.7)	172 (103.6)	<0.001
Length of stay	Mean (sd)	92 (303.9)	227 (558.7)	<0.001
Readmission	Mean (sd)	48 (330.5)	551 (9,341.6)	0.311
Reoperation	Mean (sd)	60 (552.2)	127 (783.2)	0.192
Total	Mean (sd)	1,210.7 (880.1)	1,942.8 (9,537.9)	0.149

For the 396 patients undergoing incisional hernia repair (laparoscopic n = 198, robot-assisted n = 198), the mean cost per procedure was significantly lower after robot-assisted repair (1,534 Euros vs. 2,077 Euros, *P* = 0.002, Table 5). Multivariable linear regression analysis confirmed an independent significant association between surgical approach and total procedure-related costs (robotic coeff. −583.3, CI -922.1 - -244.6, *P* < 0.001) for incisional hernia repair.

**TABLE 5:** Mean total cost per patient for robot-assisted and laparoscopic incisional hernia repair.

Variables		Robot-assisted repair (n = 198)	Laparoscopic repair (n = 198)	P
Costs (Euros)				
Robotic approach	Mean (sd)	865.3 (0)	0 (0)	<0.001
Laparoscopic approach	Mean (sd)	0 (0)	471.6 (0)	<0.001
Mesh	Mean (sd)	178 (119.3)	450 (112.5)	<0.001
Tackers	Mean [sd]	17 (60.1)	225 (46.2)	<0.001
Length of stay	Mean (sd)	228 (832.9)	555 (809.7)	<0.001
Readmission	Mean (sd)	88 (419)	199 (1,071)	0.177
Reoperation	Mean (sd)	158 (970)	178 (789.2)	0.826
Total	Mean (sd)	1,533.9 (1,584.4)	2,077 (1,840.4)	0.002

## Discussion

This nationwide database study compared the procedural costs of robot-assisted primary ventral or incisional hernia repairs with laparoscopic repairs and found the costs of a robot-assisted approach was less than traditional laparoscopic repair. In subgroup analysis, robot-assisted incisional hernia repair was found to be significantly less costly compared to laparoscopic repair, and multivariable analysis confirmed this association independently. Patients undergoing laparoscopic repair had more expensive meshes implanted, significantly longer hospital stay, and a higher rate of readmission, which added to the mean costs of the procedures. These findings suggest that a robot-assisted repair is associated with shorter hospitalization and fewer readmissions leading to a procedural cost that is less than that of a laparoscopic repair.

The result of the current study seems to confirm, that for robot-assisted hernia repair, the cost-effectiveness relies on the type of hernia. This may be because incisional hernia repair is technically more challenging compared to primary ventral hernia repair and thus a robotic approach to this patient group leads to improved outcomes. Further, the costs of the meshes used in either approach was significantly greater in laparoscopic repair, which most often was due to more expensive coated meshes placed intraperitoneal as compared to non-coated meshes placed preperitoneal or retromuscular in robot-assisted repairs. Placement of the mesh in the preperitoneal or retromuscular plane may be the technically more challenging and is easier facilitated by a robot-assisted approach. Placement of a non-coated mesh in the preperitoneal or retromuscular position is possible laparoscopically and would decrease the cost of the laparoscopic procedure, however, the data reflects that it is rarely done in Denmark, possibly because it is more difficult than an IPOM procedure or because of relatively high access to robotic platforms.

A nationwide analysis from the US found that robot-assisted ventral hernia repair was associated with shorter length of stay compared to both open and laparoscopic approach, but the hospital charges were significantly higher for the robotic approach [15]. Likewise, another study from the US concluded that hospital charges were 25% higher for a robot-assisted ventral hernia repair compared with a laparoscopic repair [24]. However, the reimbursement systems in Europe and US are very different and not directly comparable. A recent Brazilian study evaluated the direct costs associated with operating time, operating room personnel, and amount of medication, and it concluded that robot-assisted repairs were significantly more costly than laparoscopic repairs caused by longer operating room charges [25]. A multicenter randomized controlled trial concluded that the costs of robot-assisted and laparoscopic ventral hernia repairs were comparable; although the meshes in the laparoscopic group were more expensive, the increased operating time in the robotic group equalized the cost [26]. These differences in study outcomes illustrate the international variation and complexity of cost analyses.

Several studies conclude that the operating time is longer for robotic surgery, but a learning curve should be taken into account, and there is evidence that the operating time, as well as time for draping and docking of the robot, can be reduced significantly with increased experience [16, 27].

Interestingly, most studies investigating cost-effectiveness of the robotic platform solely focus on the direct procedure-related cost, and not on socioeconomical costs associated with readmissions, reoperations due to complications, or the costs associated with prolonged postoperative recovery such as sick leave or costs associated with obtaining assistance at home. As length of stay and risk of readmission was shorter in patients undergoing robot-assisted repair, these patients are probably more likely to return faster to work and daily activities, thereby reducing the overall societal and psychological expenses related to surgery.

The angulation of the robotic arms facilitates the minimally invasive approach to more complex repairs. In the current study we did not take the complexity of the hernia repair into account, but only matched patients on hernia type and horizontal defect size. Thus, although aiming for reducing the selection bias with propensity score matching, there may have been more complex repairs in the robotic group reflected by more performed TAR procedures than in the laparoscopic group, which would have further added to the benefit of the robotic repairs and decreased costs. Future studies are needed to clarify outcomes and cost-effectiveness of the robotic system in the largest and more complex ventral and incisional hernias.

This study is strengthened by its reliance on nationwide database data reflecting real world data with high external validity. However, there are limitations. The laparoscopic procedures performed were mainly IPOM procedures, whereas the robot-assisted procedures were various, and one may argue that these are not comparable. On the other hand, this is real-world data, and one of the advantages with the robotic system is easier placement of the mesh outside the peritoneal cavity. While this is also feasible laparoscopically, it is technically more challenging. Possibly, the laparoscopic procedures were performed by several different surgeons, and the robot-assisted surgeries were performed by a smaller group of hypothetically more experienced surgeons. On the other hand, the first robot-assisted procedures were included in this study, meaning that the study included cases in the early phase of the robotic learning curve, both factors could potentially have affected the results. The operative time is unfortunately not available from the Danish Hernia Database, which would have been relevant to include in the cost calculation. As robotic procedures, especially in the beginning of the learning curve, may take longer than laparoscopic procedures, calculation of cost associated to operative time could have affected the results.

Comprehensive cost calculations are difficult to perform and compare, as they vary across countries with different reimbursement systems, and inherently are based on the elements included in the computations. This cost calculation was based on the Danish system, which may not translate to other healthcare systems. A factor like length of stay may not be related to the procedure itself but to discharge traditions, patient-related factors and regional factors, as more laparoscopic repairs were performed in the early part of the study period, such factors could potentially have biased the results. In contrast, when a novel surgical procedure is implemented, such as robot-assisted ventral hernia repair, the surgeon may opt to keep the patient hospitalized for an additional day.

This cost analysis excluded expenses related to acquisition of the robotic platform and operating times, potentially underestimating the costs in the robotic group. Acquisition and maintenance costs may be attributed to different resources in the hospital (i.e., acquisition costs vs. daily expenses) and further, that the price of a robotic system may vary due to differences in hospital relations to the robotic system manufacturer or previous acquisitions in the hospital. Lastly, many hospitals already have a robotic system, but the system is not used for abdominal wall surgery, these findings suggest that if a system is present, it seems cost-effective to use it for ventral and incisional hernias [28]. Presumably, the price of acquiring a robot system is likely to change in the future, as more robotic systems are on the market and different agreements such as leasing or pay-per-procedure can be made with the companies. Furthermore, the study employed minimum prices for hospital stay, cost of readmissions and surgical reinterventions. Costs associated with further diagnostic work-up or interventions of readmitted patients, such as imaging diagnostics or epidural anesthesia, were not included in the cost calculation. Additionally, specific surgical equipment such as vacuum assisted closure (VAC), staplers or energy devices were not included in the cost of surgical reintervention, potentially impacting the overall costs.

In conclusion, while the costs of the robotic surgical equipment surpass that of conventional laparoscopy, it is offset by the need of more expensive meshes and tacker devices, and higher readmission rates following a laparoscopic approach. This nationwide database study showed that for primary ventral hernias, the mean procedural costs of a robot-assisted and a laparoscopic repair are comparable, but for incisional hernia repairs the mean procedural cost is decreased with a robot-assisted approach. Future cost analyses should be more comprehensive and include socioeconomic factors related to the postoperative phase to map the scope of robot-assisted hernia repair and its suggested benefits.

## Data Availability

The datasets presented in this article are not readily available because due to danish law, the dataset can’t be shared. Requests to access the datasets should be directed to nadiahenriksen@gmail.com.
