# Supplementary material for: Procedural Costs of Robot-Assisted and Laparoscopic Ventral and Incisional Hernia Repair. A Propensity-Score Matched Nationwide Database Study
Source: J Abdom Wall Surg. 2025 Nov 4;4:15464. doi: 10.3389/jaws.2025.15464 (PMC12623262; doi:10.3389/jaws.2025.15464)
Supplement: Supplementary file 1 [file Supplementaryfile1.docx]

**Supplementary tables**

**Reoperation specifications**

|  |  | **Robot-assisted approach (n=554)** | **Laparoscopic repair (n=554)** | **Total (n=1108)** | **P** |
| --- | --- | --- | --- | --- | --- |
| **Reoperation** | *No reoperation* | 543 (98.0) | 533 (96.2) | 1076 (97.1) | 0.248 |
|  | *Colonoscopic polypectomy* | 1 (0.2) | 2 (0.4) | 3 (0.3) |  |
|  | *Incisional hernia repair* | 1 (0.2) | 0 (0.0) | 1 (0.1) |  |
|  | *Umbilical hernia repair* | 1 (0.2) | 0 (0.0) | 1 (0.1) |  |
|  | *Other reoperation* | 1 (0.2) | 0 (0.0) | 1 (0.1) |  |
|  | *Umbilical hernia repair with mesh* | 2 (0.4) | 0 (0.0) | 2 (0.2) |  |
|  | *Lap. incisional hernia repair* | 2 (0.4) | 2 (0.4) | 4 (0.4) |  |
|  | *Excision of pathological tissue of the abdominal cavity* | 1 (0.2) | 0 (0.0) | 1 (0.1) |  |
|  | *Laparoscopy* | 1 (0.2) | 2 (0.4) | 3 (0.3) |  |
|  | *Lap. operation for adhesions* | 1 (0.2) | 0 (0.0) | 1 (0.1) |  |
|  | *Lap. cholecystectomy* | 0 (0.0) | 1 (0.2) | 1 (0.1) |  |
|  | *Excision of pathological tissue from the abdominal wall* | 0 (0.0) | 1 (0.2) | 1 (0.1) |  |
|  | *Laparotomy* | 0 (0.0) | 4 (0.7) | 4 (0.4) |  |
|  | *Reoperation for deep SSI* | 0 (0.0) | 1 (0.2) | 1 (0.1) |  |
|  | *Reoperation for hemorrhage* | 0 (0.0) | 1 (0.2) | 1 (0.1) |  |
|  | *Incision of abscess* | 0 (0.0) | 1 (0.2) | 1 (0.1) |  |
|  | *Lap. Inguinal hernia repair* | 0 (0.0) | 2 (0.4) | 2 (0.2) |  |
|  | *Percutaneous drainage of abdominal cavity* | 0 (0.0) | 2 (0.4) | 2 (0.2) |  |
|  | *Lap. umbilical hernia repair* | 0 (0.0) | 1 (0.2) | 1 (0.1) |  |
|  | *Lap. hemicolectomy* | 0 (0.0) | 1 (0.2) | 1 (0.1) |  |
| **Number of reoperations** | *0* | 543 (98.0) | 533 (96.2) | 1,076 (97.1) | 0.043 |
|  | *1* | 7 (1.3) | 19 (3.4) | 26 (2.3) |  |
|  | *2* | 4 (0.7) | 2 (0.4) | 6 (0.5) |  |

**Robotic equipment used per procedure**

| **Item** | **Price pr. item (€)** | **Comment** | **Total (€)** |
| --- | --- | --- | --- |
| Instrument drape | 201.5 | Drape for four arms. One set used per procedure | 201.5 |
| Blunt obturator | 8.07 | One per procedure | 8.07 |
| Cannula seal | 17.4 | Three used per procedure | 52.3 |
| Tip cover | 19.4 | One per procedure | 19.4 |
| Monopolar curved scissor | 2790 | Price per procedure (usable for 10 procedures) | 279.0 |
| Fenestrated bipolar forceps | 2305.8 | Price per procedure (usable for 14 procedures) | 164.7 |
| Mega suture needle driver | 2104.5 | Price per procedure (usable for 15 procedures) | 140.3 |
| ***Total*** |  |  | 865.27 |

**Laparoscopic equipment used per procedure**

| **Item** | **Price pr. item (€)** | **Comment** | **Total (€)** |
| --- | --- | --- | --- |
| Stepport | 234 | Estimated to be used in 1/3 of all procedures | 234* |
| Standard port setup¶ | 65.9 | Estimated to be used in 2/3 of all procedures | 44.0 |
| Lap. scissor | 45.8 | Once each procedure | 45.8 |
| Lap. smoke filter | 12.9 | Once each procedure | 12.9 |
| Thunderbeat | 408.7 | Every third procedure | 134.9 |
| ***Total*** |  |  | 471.6 |

*: $\frac{234 € per port \times3 ports per procedure}{Estimated use in \frac{1}{3} of all procedures}=234 €$

¶: 2 x 12 mm ports and 1 x 5 mm port

**Price per mesh**

| **Mesh name** | Cost (€) |
| --- | --- |
| Adhesix, BARD | 129.9 |
| Dynamesh | 636.4 |
| Galmesh_Light | 129.9 |
| Gore Dualmesh | 494 |
| Optilene Mesh | 85.9 |
| Parietene, Covidien | 325 |
| Parietex Composite (inkl. Optimized), Covidien | 494 |
| Physiomesh, Ethicon | 494 |
| Prolene | 312 |
| SoftMesh, BARD | 129.9 |
| Symbotex Composite | 494 |
| Ultrapro Advanced | 238.6 |
| Ventralight ST, BARD | 494 |
| Versatex | 99.6 |

**Commercial mesh name according to type of hernia repair**

|  |  | **Laparoscopic repair** | **Robot-assisted repair** | **Total** |
| --- | --- | --- | --- | --- |
| **Mesh name** | Versatex | 1 (0.2) | 17 (3.1) | 18 (1.6) |
|  | Ventralight ST, BARD | 46 (8.3) | 0 (0.0) | 46 (4.2) |
|  | Ultrapro Advanced | 1 (0.2) | 20 (3.6) | 21 (1.9) |
|  | Symbotex Composite | 264 (47.7) | 29 (5.2) | 293 (26.4) |
|  | SoftMesh, BARD | 1 (0.2) | 11 (2.0) | 12 (1.1) |
|  | Prolene | 40 (7.2) | 2 (0.4) | 42 (3.8) |
|  | Physiomesh, Ethicon | 4 (0.7) | 0 (0.0) | 4 (0.4) |
|  | Parietex Progrip, Covidien | 34 (6.1) | 455 (82.1) | 489 (44.1) |
|  | Parietex Composite (incl. Optimized), Covidien | 107 (19.3) | 0 (0.0) | 107 (9.7) |
|  | Parietene, Covidien | 35 (6.3) | 1 (0.2) | 36 (3.2) |
|  | Optilene Mesh | 2 (0.4) | 5 (0.9) | 7 (0.6) |
|  | Gore Dualmesh | 2 (0.4) | 1 (0.2) | 3 (0.3) |
|  | Galmesh_Light | 11 (2.0) | 0 (0.0) | 11 (1.0) |
|  | Dynamesh | 4 (0.7) | 0 (0.0) | 4 (0.4) |
|  | Adhesix, BARD | 2 (0.4) | 13 (2.3) | 15 (1.4) |
